# The Plague

**Published:** 1901-07-06

**Authors:** 


### THE PLAGUE.

The announcement made last week that several deaths had occurred at Oporto, although it has since been denied from Madrid, again draws attention to the march of that disease, and to the possible dangers involved in its propinquity. Let it not be forgotten that although we have been successful in suppressing a small outbreak in Glasgow, and in beating off several slight incursions of the disease at other ports, the efforts which have been made to drive plague out of the various places in which it has once obtained a footing, have been singularly unsuccessful. When eight years ago plague broke out in Hong-Kong, the seriousness of the matter was recognised, and great efforts were made to stamp it out; and when the epidemic soon died away it was hoped that we had been successful. Those, however, who were acquainted with the natural history of the disease, shook their heads. They knew that this rapid recession is just one of this malady's little ways, and they felt sure that it would show itself again in due season—as in fact it did. During the last eight years Hong-Kong has had seven definite outbreaks, and, instead of decreasing in virulence it would seem that the present one is the most fatal of the lot. This is an object lesson which must not be overlooked when we find people talking glibly about the efficacy of our sanitary measures, and the extent to which we have brought this disease under subjection. As a fact, wherever it has thoroughly taken root, there it has cropped up again and again notwithstanding all that has been done. Take the case of Bombay. Till six or seven years ago the inhabitants of Bombay thought themselves as little likely to be decimated by plague as we do in London, and when the enemy came they were perfectly lavish in the measures taken to drive it out. They may not have been altogether judicious, but they spared no effort, and to tell the truth they spared no tyranny, to effect their purpose. But plague is still endemic in Bombay, and, if we are to read the future by aid of history, there it will remain. It is now many months since plague broke out at Capetown, and considering the conditions which had been allowed to grow up there, the people of that town have every reason to be thankful for the comparatively limited extent of the mischief that has so far been caused. But notwithstanding its moderate dimensions, the epidemic still goes on, and by the extent to which it has fallen upon the European population shows—what, again, we might know well from history—that the whites possess no racial immunity to the disease. The moral of all this is that we should maintain in constant readiness the full machinery necessary for dealing with the disease, and should not hesitate to act with vigour—even with such a degree of vigour as to raise many protests—on the occurrence of the first inkling of an outbreak. In regard to no other disease is it so true as it is in reference to plague that "a stitch in time saves nine." Meanwhile, how are we getting on in suppressing the known predisposing influences—the rats, the bugs, the fleas, and all the biting parasites which dwell in companionship with gregarious man? Not very quickly, we fear.

